# Functional Assays and Metagenomic Analyses Reveals Differences between the Microbial Communities Inhabiting the Soil Horizons of a Norway Spruce Plantation

**DOI:** 10.1371/journal.pone.0055929

**Published:** 2013-02-13

**Authors:** Stéphane Uroz, Panos Ioannidis, Juliette Lengelle, Aurélie Cébron, Emmanuelle Morin, Marc Buée, Francis Martin

**Affiliations:** 1 INRA, UMR 1136 INRA, Université de Lorraine “Interactions Arbres Micro-organismes”, Centre INRA de Nancy, Champenoux, France; 2 INRA UR 1138 “Biogéochimie des Ecosystèmes Forestiers”, Centre INRA de Nancy, Champenoux, France; 3 LIMOS, UMR 7137 CNRS, Université de Lorraine, Faculté des Sciences, BP70239, Vandoeuvre-les-Nancy cedex, France; Argonne National Laboratory, United States of America

## Abstract

In temperate ecosystems, acidic forest soils are among the most nutrient-poor terrestrial environments. In this context, the long-term differentiation of the forest soils into horizons may impact the assembly and the functions of the soil microbial communities. To gain a more comprehensive understanding of the ecology and functional potentials of these microbial communities, a suite of analyses including comparative metagenomics was applied on independent soil samples from a spruce plantation (Breuil-Chenue, France). The objectives were to assess whether the decreasing nutrient bioavailability and pH variations that naturally occurs between the organic and mineral horizons affects the soil microbial functional biodiversity. The 14 Gbp of pyrosequencing and Illumina sequences generated in this study revealed complex microbial communities dominated by bacteria. Detailed analyses showed that the organic soil horizon was significantly enriched in sequences related to Bacteria, Chordata, Arthropoda and Ascomycota. On the contrary the mineral horizon was significantly enriched in sequences related to Archaea. Our analyses also highlighted that the microbial communities inhabiting the two soil horizons differed significantly in their functional potentials according to functional assays and MG-RAST analyses, suggesting a functional specialisation of these microbial communities. Consistent with this specialisation, our shotgun metagenomic approach revealed a significant increase in the relative abundance of sequences related glycoside hydrolases in the organic horizon compared to the mineral horizon that was significantly enriched in glycoside transferases. This functional stratification according to the soil horizon was also confirmed by a significant correlation between the functional assays performed in this study and the functional metagenomic analyses. Together, our results suggest that the soil stratification and particularly the soil resource availability impact the functional diversity and to a lesser extent the taxonomic diversity of the bacterial communities.

## Introduction

Soil is a complex environment inhabited by a wide range of eukaryotic, prokaryotic and viral organisms. However, little is known about the diversity, functions and interactions of these organisms. It is well established that soil microorganisms actively participate in nutrient cycling through organic matter degradation [1], nitrogen cycling and mineral weathering, thus providing plants with essential nutrients [2]–[3]. Most of this knowledge was generated using cultivation-dependent as well as -independent approaches that generally targeted only one type of organism (i.e., fungi or bacteria), consequently yielding only a partial understanding of the microbial soil assemblage. Notably, many of these studies showed that the functional and taxonomic diversity of soil microbial communities are strongly impacted by environmental factors such as edaphic characteristics (pH, nutrient availability), climatic modifications and/or biotic interactions [4]–[7], suggesting that the soil microbial diversity may be considered good indicator of how the ecosystem is functioning.

In the last decade, pyrosequencing and Illumina-based surveys have been performed to follow this indicator and are now commonly used to determine the diversity and the distribution of the microbial communities in different environments, including grassland [8]–[9], farmland [10] and forest soils [11]–[15], sea sediments [16]–[18], and mine biofilms [19]. In addition to these surveys, a recent study has combined DNA and RNA based tag-encoded amplicon pyrosequencing for the first time to analyse the diversity of the microbial communities as well as the active microorganisms in the upper layers of the soil in a spruce (*Picea abies*) forest [20]. This approach revealed that a potential gap exists between the presence and the activity of soil microorganisms and showed that low-abundance species could be highly active in the soil. However, although these studies produced a comprehensive view of the microbial communities based on the sequencing of marker genes such as 16S rRNA or nuclear ribosomal internal transcribed spacer (ITS) sequences, none of them examined the potential functions of the microbial communities.

In addition to determining the diversity and distribution of microbial communities in an environment using tag-encoded amplicon pyro- or Illumina-sequencing, direct shotgun sequencing-based analyses provide new methods to explore the functional potentials of these complex communities and to discover novel functions. This approach was recently used to establish a catalogue of the microbial genes present in the human gut [21], or associated with leaf-cutter ants [22] and explore the metagenome of terrestrial and marine environments [14], [16], [23]–[25]. Most recently, Fierer *et al*. [4], Delmont *et al*. [26] and Mackelprang *et al*. [27] applied shotgun metagenomics to grassland, agricultural soils and permafrost to characterise the impact of fertilisation, seasonal changes, the vertical distribution and the response to thaw on the soil microbial communities. These shotgun post-analyses were made possible by the development of several bioinformatics tools that can be used to (i) manage the flow of generated data, (ii) compare data with specialised databases (i.e., CAZYmes [28]; MG-RAST [29]; RDP [30]), (iii) extract relevant information and (iv) open new perspectives for our understanding of the soil microbiome.

The main objectives of this study were to determine how the structure and functional abilities of the soil microbial communities are impacted by the soil forest stratification. We hypothesised that the natural decrease of available nutrients between the organic and mineral horizons as well as the difference of pH would impact the distribution of the microbial communities. To test this hypothesis, we considered the Breuil-Chenue long-term observatory (LTO), for which we have culture-dependant information related to the functional potentials of the microbial communities [31]–[33] and culture-independant information related to the distribution of the microbial communities according to their habitat location [7], [12], [34]–[35]. In order to obtain the most comprehensive view of the forest soil microbial communities, we applied in this study shotgun pyrosequencing and Illumina-based DNA sequencing to soil core replicates from a temperate and non-amended forest site (Breuil-Chenue, France). The biological replicates were collected under a Norway spruce (*Picea abies*) stand, considering the organic (Org-S) and mineral (Min-S) soil horizons, two horizons characterised by their different nutrient availability, C quality/quantity and root density. Because of the importance of organic matter degradation in forest soils, we focused our analysis on how carbohydrate-active enzymes (CAZymes) were distributed in these two horizons. The same soil samples were also used to quantify the fungal and bacterial communities and to determine the functional potentials of these microbial communities using enzymatic and metabolic assays.

## Materials and Methods

### Ethics Statement

No specific permits were required for the described field studies. The study location is not privately owned or protected in any way and the field studies did not involve endangered or protected species.

### Site Description, Sampling and Soil Characterisation

The Breuil-Chenue long-term observatory (LTO) is located in the Morvan (47°18′N, 4°5′E, France). The native forest was partially clear-cut and replanted in 1976 with mono-specific tree plantations distributed in 0.1-ha plots of different species, including the Norway spruce (*Picea abies*). The soil is an Alocrisol that developed on the Pierre qui Vire granite. To avoid horizontal spatial heterogeneity and to focus our analysis on the potential vertical differentiation, a small-scale sampling strategy was applied. Three adjacent soil cores (5×5×20 cm; length × width × depth, without the litter layer), distant of 20 cm, were sampled in March 2010 under the Norway spruce trees. The separation of the organic layer (0–10 cm) and the mineral layer (10–20 cm) was performed in the lab for each biological replicate (soil core). The soil samples were sieved (2 mm mesh) and homogenised prior to enzymatic and molecular analyses for each replicate, while replicates were pooled for soil analyses.

### Enzymatic Assays

To compare the functional potentials of the soil microbial communities inhabiting the organic and mineral horizons, we used a combination of metabolic and enzymatic bioassays. Briefly, 5 g of soil from each soil sample were shaken in 45 ml sterile water on an orbital shaker at 25°C. A 10^−1^ dilution of each soil sample was then used to inoculate Ecoplate microplates (BIOLOG^®^). The plates were incubated at 25°C, and colour development was measured at 590 nm with a microplate reader (Bio-Rad model 550) after a 48 h incubation period. Furthermore, six enzymatic activities based on a short incubation time were measured using 2,2′-azinobis-3-ethylbenzothiazoline-6-sulfonate (ABTS) as a substrate for laccase activity and five derivatives of methylumbelliferone as substrates to measure the acid phosphatase, ß-glucosidase, exochitinase, xylosidase and cellobiohydrolase activities; these methods were adapted from Pritsch *et al*. [36]. All of the chemicals were purchased from Sigma-Aldrich (France). For the laccase test, a solution of 2 mM ABTS was prepared in sodium acetate buffer at pH 4.5. In each well of a clear flat-bottom 96-well plate (Sarstedt, Newton, NC, USA), 75 µl of the diluted soil solution (1∶10) was added to 75 µl of 2 mM ABTS solution. After incubation at 25°C, measurements were performed at 415 nm. For the microplate assays with the methylumbelliferone substrates, 50 µl of the diluted soil solution, 50 µl of incubation buffer (Sodium acetate 100 mM, pH 4.5), and 50 µl of the substrate solution were added to each well. After incubation, 100 µl of stopping buffer (Tris 2.5 M pH 10–11) was added to the reactions. Measurements were carried out using a Victor^3^ microplate reader (Wallac Perkin-Elmer Life Sciences, Villebon-sur-Yvette, France) with an excitation wavelength of 360 nm and an emission wavelength of 450 nm.

### DNA Extraction Methods

To obtain the required amount of metagenomic DNA (mgDNA) (30 µg per sample) and limit the DNA extraction bias [37]–[38], mgDNA was extracted from 10 g of each soil sample using two independent DNA extraction procedures. To obtain a comprehensive view and to avoid erroneous conclusions, independent biological replicates were treated for each soil horizon [39]. The first procedure was based on the MoBio PowerMax Soil DNA isolation kit (MoBio Laboratories, Carlsbad, CA, USA) with the addition of proteinase K (50 mg ml^−1^) and lysosyme (10 mg ml^−1^) in the first step. In the second procedure, mgDNA was extracted from soil samples using a FastDNA Spin Kit for Soil (MP Biomedicals, Solon, OH, USA) with the addition of 20 mg of Polyvinyl-polypyrrolidone (Sigma-Aldrich, France) to each soil sample before the grinding step. The quantity and quality of the mgDNA were evaluated by agarose gel electrophoresis and with a Nanodrop 1000 spectrophotometer (Thermo Scientific, Wilmington, DE, USA). The mgDNA extracted by these two methods were then pooled to obtain 30 µg of mgDNA for each biological replicate, resulting in 3 organic mgDNA samples (Org-S; O1 to O3) and 3 mineral mgDNA samples (Min-S; M1 to M3). The soil replicates were sequenced using a half-plate 454 pyrosequencing run (about 500,000 reads per replicate) and one Illumina lane (about 30 million reads per replicate). Pyrosequencing on the Genome Sequencer (GS) FLX Titanium 454 System (Roche) at Beckman Coulter Genomics (Beverly, MA, USA) resulted in ca 6.13×10^6^ reads that passed the length and quality criteria. Sequencing on 2 lanes of the HiSeq 2000 (Illumina) was performed by Beckman Coulter Genomics (Danvers, MA, USA) and produced 193×10^6^ filter-passed reads.

### Real-time PCR Quantification of Total Bacteria and Fungi

The mgDNA was used to quantify the total bacterial and fungal communities using 16S and 18S rRNA gene-specific primers (968F/1401R and Fung5f/FF390r, respectively; [40]–[41]). The real-time PCR quantifications were performed in triplicate as described in Cébron *et al*. [42] and Thion *et al*. [43], using an iCycler iQ apparatus associated with optical system interface software (version 2.3; Bio-Rad). Amplification reactions were carried out on 1 µl of template mgDNA, standard plasmids (10^8^ to 10^2^ gene copies·µl^−1^) or water (negative control), in a volume of 20 µl using iQ SYBR green Supermix (Bio-Rad). Amplification temperature profiles consisted of: 5 min at 95°C, followed by four steps of 50 cycles, 30 s at 95°C, 30 s at the primer-specific annealing temperatures (56°C and 50°C for 16S and 18S rRNA, respectively), 30 s at 72°C, and 10 s at 80°C to dissociate the primer dimers and capture the fluorescence intensity of the SYBR green. Amplification efficiencies were of 87.2 and 85% for 16S and 18S amplification, respectively. At the end, a melting curve analysis was performed from 50°C to 95°C, with a temperature increase of 0.5°C every 10 s. Melting temperature was 89°C for 16S amplicons for both horizon samples and 85.0°C and 85.5°C for 18S amplicons from mineral and organic horizons, respectively. The presence of PCR inhibitors was evaluated by mixing 1 µl of environmental DNA with 1 µl of 10^5^ copies of lambda-standard plasmid and compare to lambda-standard curve as described in Cébron *et al*. [42].

### MG-RAST Analysis

Raw sequences for each horizon along with the corresponding quality values were uploaded to the MG-RAST server [29]. For the pyrosequencing sequences, the taxonomic analysis was performed using two databases: Ribosomal Data Project (RDP) (an e-value threshold of 0.01 and a minimum percentage identity of 80%) for the 16S rRNA fragments identified, and SEED (an e-value cut-off of 1e−05) for all the other sequences. The default parameters of the MG-RAST were used for the taxonomic and functional assignation of the Illumina sequences. All of the Illumina reads that were shorter than 35 bases or had a median quality score below 20 were removed.

### Carbohydrate-active Enzymes: Annotation and Analysis

Raw sequences of the Breuil-Chenue metagenomes (pyrosequencing reads) were searched for carbohydrate active enzymes (CAZymes) using the CAZY database [28] reference dataset found at the CAT (CAZymes Analysis Toolkit) web service [44]. The assignation was searched against this reference dataset using protein BLAST with an e-value cut-off of 1e−05.

### Statistical Analyses

The effect of the origin of the soil suspensions on the enzymatic assays was determined by analysis of variance (one factor ANOVA). Quantification data from real-time PCR were compared as Log gene copy number between the two soil horizons through a Student-t test. To determine the taxonomic, metabolic and CAZyme differences between the organic and mineral metagenomes extracted from MG-RAST, one-factor (horizon origin) ANOVA at a threshold level of P = 0.05 and a Bonferroni-Dunn test were applied on the relative distribution values after an arcsine transformation. All statistical analyses were performed using the Superanova software (Abacus Concepts, Inc., Berkeley, CA). Taxonomic (based on the genera assigned in MG-RAST; H’_T_), functional (based on the functional categories assigned in MG-RAST; H’_F_) and metabolic diversity (based on the Biolog profiles; H’_m_) levels per biological replicate were estimated using the Shannon index (H’). Mantel tests (10,000 permutations, Pearson correlations) were performed in XLstat2011 (Addinsoft, Paris, France) in order to test correlations between biolog, metagenomic functions and SEED genera, based on Euclidean distances dissimilarity matrices generated from scaled data.

### Nucleotide Sequence Accession Numbers

The sequences generated in this study have been deposited on the Sequence Read Archive (SRA) service of the GenBank database under the accession numbers SRA055292 (for the Illumina based shotgun soil metagenomic biological replicates), and SRA055323 (for the 454 based shotgun soil metagenomic biological replicates).

## Results and Discussion

### Soil Characteristics, Enzymatic Activities and Microbial Quantification

**Figure 1:**
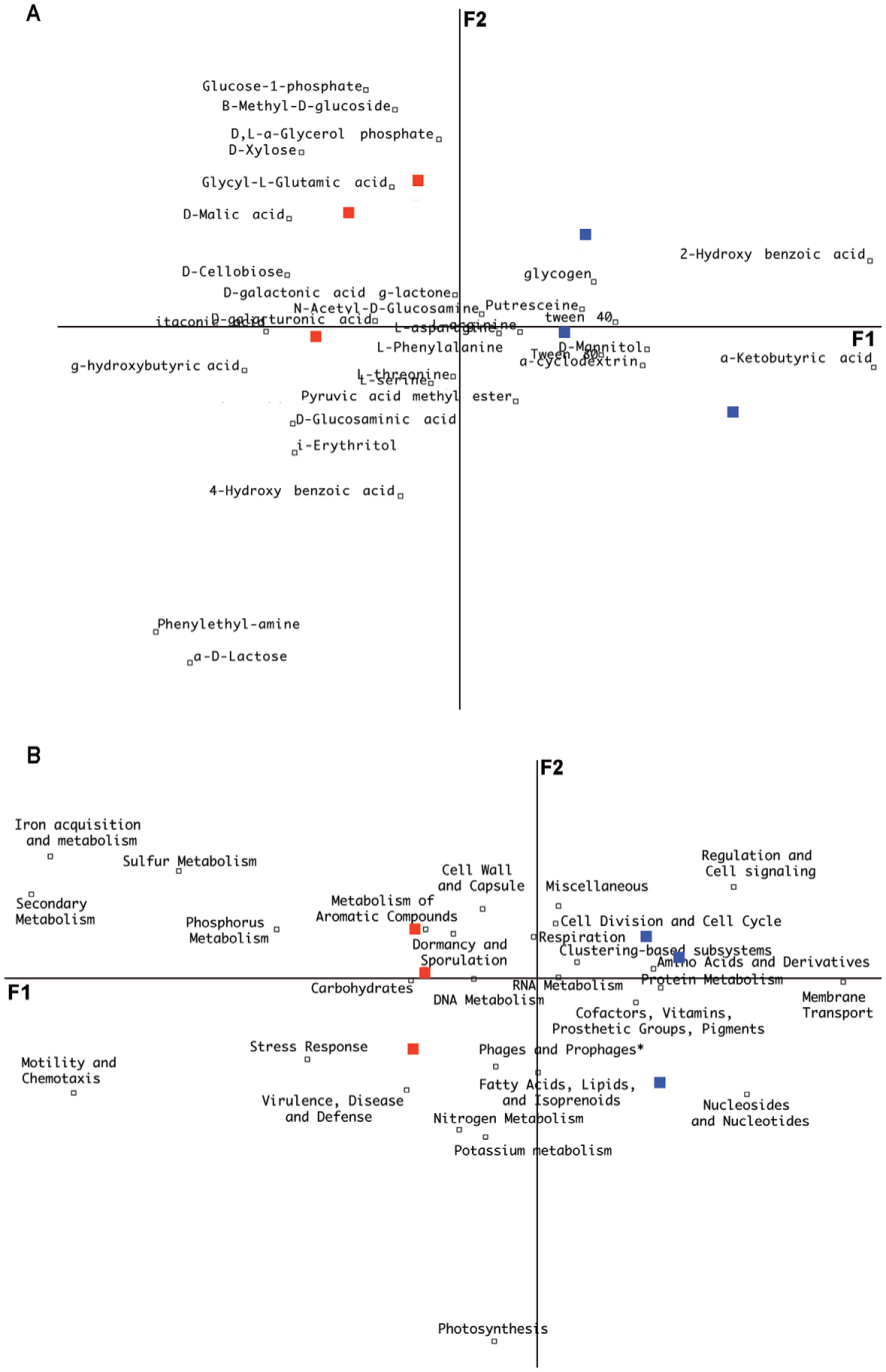
Multifactorial analysis. A. Analysis of the metabolic patterns based on the Biolog Ecoplate ® **results.** In this analysis, principal component axis 1 and 2 explain most of the variance in the data cumulatively (F1 = 37.3% and F2 = 21.3%). **B. Analysis of the relative proportion of the major functional categories present in the metagenomic data of the organic and mineral soil horizons.** In this analysis, principal component axis 1 and 2 explain most of the variance in the data cumulatively (F1 = 72% and F2 = 8.89%). Soil metagenomes are presented as follow: red squares, biological replicates from the organic horizon; blue squares, biological replicates from the mineral horizon.

Soil analyses revealed that the organic soil horizon was more acidic (pH = 4) than the mineral horizon (pH = 4.6) and was characterised by a high level of organic and inorganic nutrients (Table 1), confirming results obtained in previous studies on the same experimental site [32], [45]. Metabolic assays performed using Biolog Ecoplates® on the soil replicates revealed significant differences between the two soil horizons. The organic horizon samples were characterized by a significantly higher metabolic based Shannon diversity index than the mineral horizon samples (metabolic diversity; H_m-organic_ = 2.96±0.03 and H_m-mineral_ = 2.47±0.08; P = 0.01). Among the 31 carbon sources included in the Biolog Ecoplates®, 20 substrates related to carbohydrates, amino acids, cellulose and chitin derivatives were significantly more metabolised in the organic horizon (*P*<0.05) (Table S1). A multivariate analysis confirmed the differentiation of the two soil horizons according to their metabolic profiles (Figure 1A). Although the metabolic capabilities of the communities residing in the organic soil horizon were significantly higher in our experimental conditions than those of the mineral soil horizon, the most intensively metabolised substrates were different between the two horizons. L-asparagine was the most intensively metabolised substrate in the organic horizon, whereas D-mannitol was the most intensively metabolised substrate in the mineral horizon, suggesting a specialisation in response to the nutritional conditions. Similarly, all of the enzymatic assays showed that the laccase, phosphatase, glucosidase, exochitinase, xylosidase and cellobiohydrolase activities detected in the soil solutions were significantly higher in the organic soil horizon (*P*<0.05). Interestingly, Snajdr *et al*. [46] also reported spatial variability of enzyme activities in the upper layers of *Quercus petraea* forest soil as well as modification of the microbial communities. Taken together, these data suggest that the microbial communities inhabiting the organic horizon are specialised to exploit its relatively rich and complex substrates compared to those of the mineral horizon.

**Table 1:** Soil characteristics of the Norway spruce plantation of the Breuil-Chenue forest.

Soil horizon	pH	N	C	P	CEC*	Ca	Mg	K
	g kg^−1^				cmol_c_ kg^−1^			
Mineral	4.63	1.41	26.4	0.11	9.66	nd‡	0.042	0.1
Organic	4.01	5.98	113	0.19	26.5	0.546	0.425	0.4

* CEC : Cation exchange capacity.

‡ nd: non detectable.

Quantification of the total bacterial and fungal communities using real time PCR demonstrated that significantly more bacteria were detected in the organic horizon (4×10^9^±9.7×10^8^ average gene copy number of 16S rRNA per g of soil) than in the mineral horizon (2×10^9^±2.4×10^8^ average gene copy number of 16S rRNA per g of soil) (*P* = 0.016). Similarly, significantly more fungi were detected in the organic horizon (4.3×10^7^±1.1×10^4^ average copy number of 18S rRNA per g of soil) than in the mineral horizon (9.5×10^6^±3×10^3^ average copy number of 18S rRNA per g of soil) (*P* = 0.0003). Baldrian *et al*. [20] reported a decrease in the fungal abundance in the organic horizon compared to the litter, and with our present study, this suggests that the fungal abundance decreases from the litter to the mineral horizon. Using 454 sequencing of the fungal ITS region, Jumpponen *et al*. [47] reported also that community richness and diversity estimators tended to decrease with increasing soil depth. The ratio of the abundance of fungi relative to the abundance of bacteria was very low (R_mineral_ = 4.5×10^−3^ and R_organic_ = 1.05×10^−2^), indicating that the bacteria were dominant in the ssu rRNA gene pool. Similarly, Will *et al*. [9] demonstrated that the microbial biomass was greater in the organic horizon than in the mineral horizon. These quantitative differences likely explain the higher enzymatic activities measured in the organic horizon.

### Taxonomic Analyses

**Figure 2:**
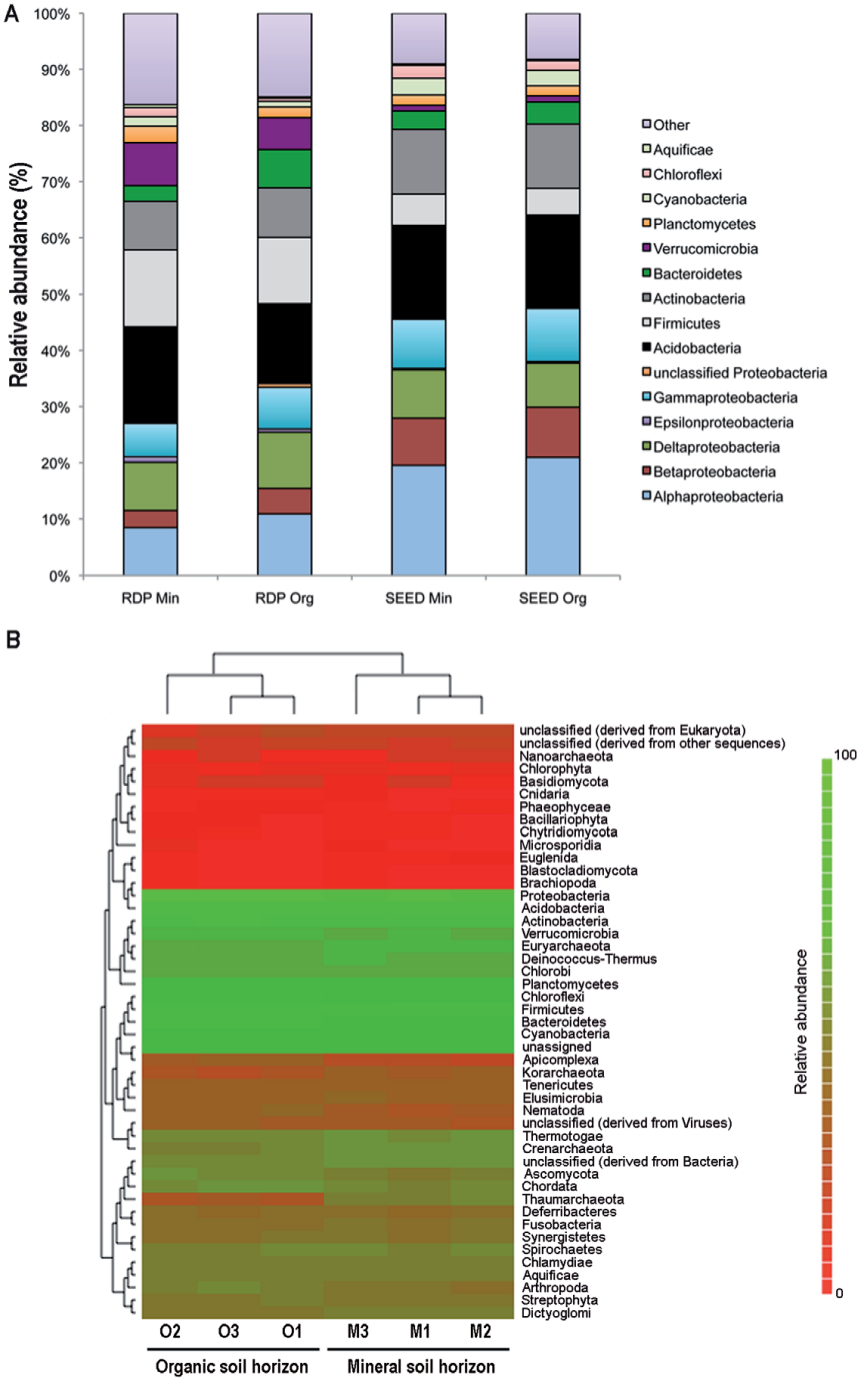
Distribution of phylogenetic groups in forest soil organic (ORG-S) and mineral (Min-S) horizons. A. RDP and SEED-bases analysis. The distribution of the reads and contigs generated in this study was performed using all the annotated reads against the SEED database and the identified 16S rRNA fragments against the RDP database. **B. Community comparison.** The phylogenetic heatmap presented was performed using the Bray-Curtis similarity matrices generated by MG-RAST on the organic (O1,O2 and O3) and the mineral (M1,M2 and M3) metagenomic data.

Metagenomic DNA extracted from each soil replicate was used to assess the structure and diversity of the soil microbial communities inhabiting the two soil horizons. According to the SEED database of the MG-RAST portal, the microbial communities inhabiting the two soil horizons were taxonomically similar (Figure 2A and 2B). This analysis also highlighted that *ca*. 53% of the pyrosequencing reads had a significant match in the nucleotide databases, which corresponded to a total of 1,064,196 reads from the Min-S samples and 1,134,455 from the Org-S samples. Of these, the vast majority of the matching reads (*ca.* 94% for each horizon) belonged to bacteria, with a significant higher proportion in the organic horizon (Table 2), supporting the quantitative results obtained by the real time PCR (Table 3). The prevalence of bacterial sequences was also reported in other shotgun metagenomic surveys that were performed on coral, mussels, swamps and sediments [14], [16], [48]–[49]. In these studies, eukaryotic sequences represented between 0.13 to 2% of the total set of assigned sequences.

**Table 2:** Comparison of the organic (O) and mineral (M) metagenomes at the domain, phylum and class levels.

DOMAIN		PHYLUM		CLASS	
O>M	M>O	O>M	M>O	O>M	M>O
*Bacteria* **	*Archaea* ***	*Proteobacteria* **	*Firmicute* **	*Alpha proteobacteria* **	*Solibacteres* *
*Eukaryota* **		*Bacteroidetes* **	*Chloroflexi* ***	*Beta proteobacteria* **	*Delta proteobacteria* ***
		*Verrucomicrobia* **	*Deinococcus-Thermus* ***	*Gamma proteobacteria* ***	*Epsilon proteobacteria* **
			*Euryarchaeota* **	*Sphingobacteria* ***	*Bacilli* **
		*Chordata* **	*Thaumarchaeota* ***	*Cytophagia* ***	*Chloroflexi* ***
			*Crenarchaeota* ***	*Opitutae* ***	*Deinococci* ***
		*Ascomycota* **	*Chlorobi* *	*Gloeobacteria* **	*Chlorobia* *
			*Thermotogae* *	*Bacteroidia* ***	*Thermomicrobia* ***
		*Arthropoda* *	*Aquificae* **	*Flavobacteria* ***	*Archaeoglobi* *
			*Dictyoglomi* ***		*Aquificae* **
			*Fusobacteria* **	*Sordariomycetes* ***	*Thermotogae* ***
					*Clostridia* **
				*Insecta* *	*Fusobacteria* **
					*Dictyoglomia* ***
					*Dehalococcoidetes* ***
					*Methanococci* *
					*Thermoplasmata* **
					*Methanobacteria* **
					*Methanomicrobia* **
					*Thermococci* **
					*Thermoprotei* ***
					*Halobacteria* ***

* P<0.05,

** P<0.005,

*** P<0.0005. Identity was determined using the SEED database of MG-RAST with an e-value cut-off of 1e−05.

The relative distributions of the sequences in the different taxonomic levels considered were analyzed by one-factor ANOVA (and a Bonferroni-Dunn test, P<0.05).

**Table 3:** Summary of pyrosequencing data obtained from the soil horizons.

	Mineral				Organic			
	M1	M2	M3	Min- Pool	O1	O2	O3	Org-Pool
No. of sequences	591,141	692,571	644,810	582,413	752,773	555,170	618,135	612,119
No. of sequences after QC*	411,805	534,430	511,904	544,597	621,394	433,459	507,994	569,373
Total length of sequences (Mbp)	189	246	225.5	269	290	190	219	288.5
Average length of sequences (bp)	459±119	460±104	440±95	490±83	466±82	438±103	431±99	502±84
**Percentage of non annotated reads**	51.8	48.0	48.6	40.4	63.6	40.7	34.2	44.3
**MG-RAST-assigned reads (known function with SEED)**	198,683	277,809	262,940	324,764	226,080	257,071	334,247	317,057
Archaea	3930	6117	5028	3680	2465	2955	3707	6759
Bacteria	186,957	260,719	247,518	307,746	214,805	243,314	317,110	297,944
Eukaryota	1,270	1,716	1,652	2,901	1,928	2,492	3,008	1,918
Viruses	56	33	43	88	53	89	68	62
Other	6,470	9,224	8,699	10,349	6,829	8,221	10,354	10,374
**MG-RAST-assigned reads (RDP)**	126	155	134	170	111	213	198	157
Proteobacteria	32	41	36	50	34	79	68	54
Firmicutes	16	23	25	14	9	18	25	28
Actinobacteria	13	9	9	20	8	20	18	15
Acidobacteria	17	29	23	33	15	33	30	20
Bacteroidetes	2	5	3	7	8	28	10	3
Other Bacteria	46	48	38	46	37	35	47	37

* quality control.

A comparison of the bacterial communities at the phylum level, based on the clustering of data from Bray-Curtis similarity matrices (MG-RAST), demonstrated that the soil metagenomes were grouped according to their ecological origin (*i.e*. horizon, Figure 2B). Whatever the soil horizon considered, the five dominant bacterial taxonomic groups were Proteobacteria (45.83±0.91% for Min-S and 47.76±1.29% for Org-S), Fibrobacteres/Acidobacteria (16.37±0.37% for Min-S and 16.28±0.37% for Org-S), Actinobacteria (11.52±0.35% for Min-S and 11.46±0.36% for Org-S), Firmicutes (5.57±0.40% for Min-S and 4.74±0.45% for Org-S), and Bacteroidetes/Chlorobi (3.28±0.30% for Min-S and 3.92±0.36% for Org-S) (Figure 2A). Statistical analyses performed at the phylum or class levels revealed that Proteobacteria (alpha, beta- and gamma-Proteobacteria), Bacteroidetes (Bacteriodia, Chlorobia, Cytophagia, Flavobacteria, Sphingobacteria), and Verrumicrobia (Opitutae) were significantly enriched in the organic horizon. On the contrary, the mineral horizon appeared significantly enriched in sequences related to the Firmicutes (Bacilli,Clostridia), Chloroflexi (Dehalococcoidetes) and some other phyla (Table 2). Horizon-dependent abundance of certain phyla was also reported in several studies. Considering a grassland soil and replicate samples, Will *et al*. [9] highlighted that Bacteroidetes, Verrucomicrobia and Proteobacteria were enriched in the organic horizon and Chloroflexi in the mineral horizon, suggesting a specialisation to the relatively rich soil organic horizon. Focusing on the Verrucomicrobia, Bergmann *et al*. [8] reported that their relative abundance varied across soil profiles. Altogether, these results suggest a differential distribution of the bacterial communities across the soil profile according to the available nutritive resources of the soil, with communities adapted to easily accessible carbon substrates in the organic horizon (Bacteroidetes, Verrucomicrobia and Proteobacteria) and communities adapted to recalcitrant carbon substrates and inorganic nutrients in the mineral horizon (Firmicutes, Chloroflexi) [8]–[9]. At the genus level, the four most dominant genera were *Candidatus Solibacter* (Acidobacteria; 9.26±0.35% for Min-S and 8.94±0.27% for Org-S), *Candidatus Koribacter* (Acidobacteria; 7.10±0.04% for Min-S and 7.33±0.20% for Org-S), *Bradyrhizobium* (Proteobacteria; 3.55±0.09% for Min-S and 3.63±0.12% for Org-S) and *Burkholderia* (Proteobacteria; 3.01±0.14% for Min-S and 3.37±0.19% for Org-S). Although the taxonomic diversity (H’_T_ index based on the genera identified through SEED assignment) and the rarefaction analyses were not significantly different between the two soil horizons, significant differences were measured in the relative distribution of some bacterial genera (n = 120; Data not shown). Among the most abundant, *C. solibacter* and *Burkholderia* were significantly enriched in the mineral horizon and the organic horizon, respectively (P*_solibacter_* = 0.03; P*_Burkholderia_* = 0.001). Similarly, *Solibacter* (6.72±0.29%) and *Bradyrhizobium* (4.89±0.64%) were dominant in the grassland soil metagenomes of the experimental station of Rothamsted [26]. Notably, monogenic (16S rRNA) metagenomic studies performed on the Breuil-Chenue forest site have also showed that these bacterial genera were among the five dominant genera [7], [35]. The prevalence of these genera was also highlighted in other soils [4], [20], [50].

Regions of the 16S rRNA gene sequences were found in our metagenomic dataset and used for confirming the taxonomic distribution. A total of 573 and 667 reads (0.05% of the total reads assigned to bacteria) from the Min-S and Org-S samples, respectively, had a significant match with the 16S rRNA gene sequences in the RDP database. Although the 16S rRNA-based analyses are known to provide more accurate taxonomic information, the limited number of 16S rRNA gene fragments identified here precludes relevant statistical analyses. Low proportions (0.01 to 0.20%) of 16S rRNA gene sequences were also reported in other shotgun studies such as in biogas fermenters [51]. Notably, both classifications (RDP and SEED) gave similar results, with the exception that the Verrumicrobia and Planctomycetes were relatively more abundant in the RDP-based classification (Figure 2A). Similarly, Kanokratana *et al*. [14] and Delmont *et al*. [26] demonstrated that the taxonomic distribution obtained using only 16S rRNA gene sequences was different from that obtained using all the annotated sequences.

Regarding the other kingdoms, Archaea were significantly more abundant in the mineral horizon (1.81±0.23% of the annotated reads) than in the organic horizon (1.37±0.25% of the annotated reads) (P = 0.0003) (Table 2). Notably, the Thaumarchaeota (Figure 2B), which are for part known as potential chemolithoautotrophic ammonia-oxidisers, appeared enriched in the mineral horizon and may play a key role in nutrient cycling in forests with acidic soils [52]. In contrast, the eukaryotic reads were significantly more abundant in the organic horizon (0.83±0.08% of the annotated reads) than in the mineral horizon (0.69±0.07% of the annotated reads)(*P* = 0.001). Eukaryotic species diversity was almost the same in the two horizons, with a significant enrichment of sequences related to Chordata, Arthropoda and Ascomycota in the organic horizon (Table 2). The higher proportion of insects’ DNA sequences in the organic horizon supports previous findings, which suggested the important role of the insects and the macrofauna in organic matter decomposition in terrestrial ecosystems [1]. Aside from their direct role in nutrient cycling, many studies have now highlighted their role as drivers of the soil microbial diversity [53]. Concerning the fungi, Lindahl *et al.* [54] also reported that these communities are vertically structured in the soil. In our study, a total of 0.2% of the annotated reads had a significant match with fungi. Notably, most of the fungal reads belonged to Ascomycota according to the MG-RAST or NCBI databases. A significant enrichment of sequences related to *Sordariomycetes* was observed in the organic horizon (Table 2). These fungi are ubiquitous and most of the members of this phylogenetic class are considered as potential pathogens, endophytes and/or saprobes involved in plant organic matter decomposition and nutrient cycling. However, in this same spruce plantation, two species diversity surveys previously showed that the number of fungal species was relatively high [12], [31]. The gap between the eukaryotic data obtained by our shotgun metagenomic approach and the diversity observed in the same experimental site by other methods illustrates the need to use alternative approaches such as metatranscriptomics targeting polyA RNA [55]–[56] to study the soil eukaryotic communities. A small fraction of the reads were identified as belonging to viruses (Table 3).

### Metabolic and Functional Analyses

**Figure 3:**
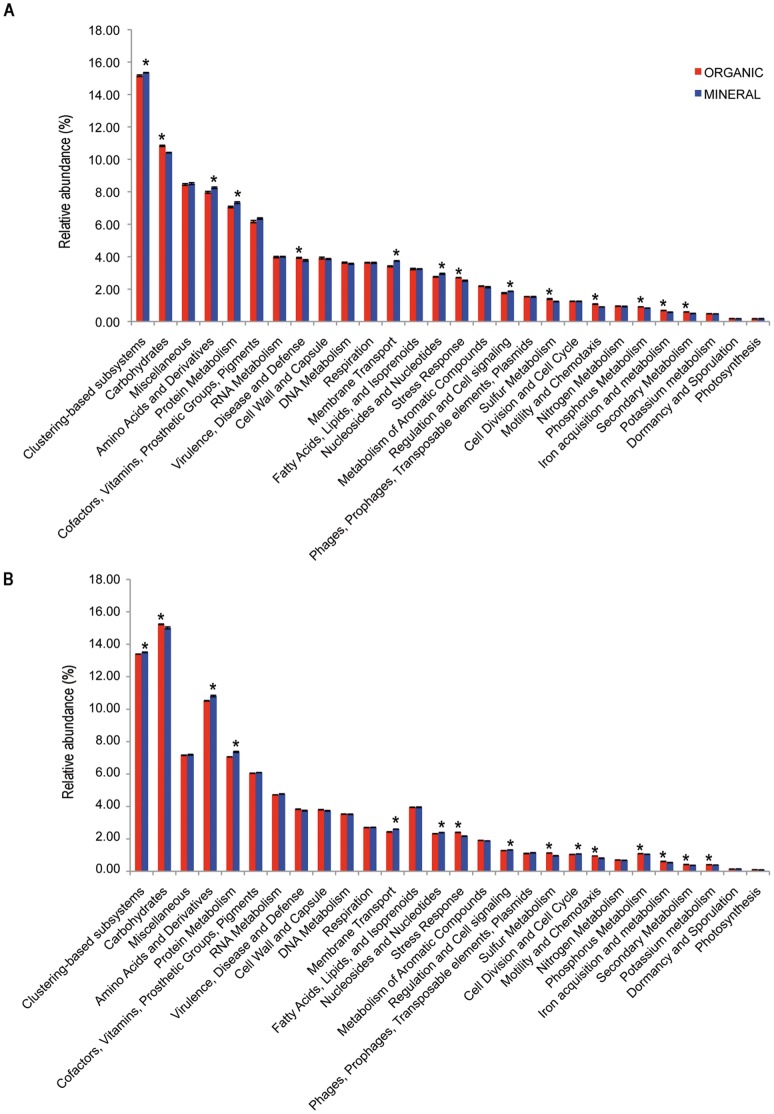
Functional analysis of the organic and mineral horizon metagenomes based on the MG-RAST database. The reads from the soil organic and mineral horizons of the forest experimental site of Breuil-Chenue (France) were screened for known functional subsystems. The data presented here corresponds to the hierarchy subsystem 1. All the data have been normalized by the quantity of assigned reads for each environment. **A. Pyrosequencing based analysis.** The presence of an asterisk indicates a significant difference (p<0.05) between the two soil horizons. The organic horizon appeared significantly enriched in sequences annotated in the carbohydrates (p<0.0001), virulence (p = 0.0256), stress response (p = 0.0013), sulfur metabolism (p = 0.0012), motility and chemotaxis (p<0.0001), phosphorus metabolism (p = 0.0001), iron acquisition and metabolism (p = 0.0002) and secondary metabolism (p = 0.0003) categories. On the contrary mineral horizon appeared significantly enriched in sequences annotated in the clustering-based subsystems (p = 0.0132), amino acids and derivatives (p = 0.0088), protein metabolism (p = 0.0012), membrane transport (p<0.0001) nucleosides and nucleotides (p = 0.0024) and regulation and cell signalling (p = 0.0142) categories. **B. Illumina-based analysis.** The presence of an asterisk indicates a significant difference (p<0.005) between the two soil horizons. The organic horizon was significantly enriched in sequences annotated in the carbohydrates (p = 0.003), iron acquisition (p = 0.005), motility and chemotaxis (p = 0.0004), phosphorus metabolism (p = 0.002), potassium metabolism (p = 0.0015), secondary metabolism (p = 0.0008), stress response (p = 0.0001) and sulfur metabolism (p = 0.0001). The mineral horizon was characterised by significantly more sequences annotated in the amino acids and derivatives (p = 0.0047), clustering-based subsystems (p = 0.0046), membrane transport (p = 0.0005), nucleosides and nucleotides (p = 0.0008), protein metabolism (p = 0.0005), regulation and cell signalling (p = 0.0045) categories.

Assignation of the reads from each soil horizon using the KEGG Mapper tool of the MG-RAST gave an integrated view of the global metabolism that revealed that most of the metabolic pathways were detected in both soil horizons (Data not shown). Only a few metabolic pathways appeared to be horizon-specific, potentially due to the insufficiency of the sequencing effort or to real biological differences. The functional differentiation of the two soil horizons was confirmed by the functional Shannon diversity index (H’_F_) calculated for the organic horizon, which was significantly higher than in the mineral horizon (P = 0.01). Among the functional categories identified by MG-RAST, the five most dominant categories based on the relative abundance of assigned reads were the clustering-based subsystems (functional coupling evidence but unknown function; 15.34±0.02% for Min-S and 15.15±0.05% for Org-S), carbohydrates (10.41±0.02% for Min-S and 10.83±0.04% for Org-S), miscellaneous (8.50±0.06% for Min-S and 8.45±0.05% for Org-S), amino acids and derivatives (8.25±0.05% for Min-S and 7.97±0.06% for Org-S) and protein metabolism (7.33±0.05% for Min-S and 7.06±0.05% for Org-S) (Figure 3A). Comparative analysis of the two soil horizons based on the full set of replicates revealed that 8 and 6 functional categories (Level 1 of MG-RAST), which corresponded to 50% of the characterised functional categories of MG-RAST, were significantly more abundant in the organic and mineral horizon, respectively (Figure 3A). The organic horizon was significantly enriched in sequences annotated in the carbohydrates category (p<0.0001). This result could be linked to the relative enrichment of spruce roots in the organic horizon that may structure the microbial communities [57]–[58]. In contrast, the mineral horizon appeared significantly enriched in sequences annotated in the clustering-based subsystems (p = 0.0132) and amino acids and derivatives (p = 0.0088). A detailed analysis (level 2 of MG-RAST) of the most abundant functional categories related to the carbohydrates in the organic horizon highlighted that the significant differences observed were mainly based on the higher abundance of annotated reads corresponding to glycoside hydrolases (p = 0.045), beta-glucuronide utilisation (p = 0.0002), organic acids (p = 0.0001) and sugar utilisation (p = 0.0002) in the organic horizon compared to the mineral horizon. The same analysis performed in the mineral horizon on the most abundant functional categories related to the amino acids and derivatives showed that the significant differences observed were mainly due to a higher abundance of annotated reads related to histidine metabolism (p = 0.0007) and proline and 4-hydroxyproline metabolism (p = 0.0118) in the mineral horizon compared to the organic horizon. Significantly more ABC transporters related to iron acquisition were observed in the mineral horizon. When compared at the finer level (level 3 of MG-RAST), the most prevalent subsystems were related to carbohydrates (ca 1.8±0.01% of the annotated reads), cofactors, vitamins, prosthetic groups, and pigments (ca 1.55±0.10% of the annotated reads). Based on these analyses, it can be concluded that the microbial communities inhabiting the organic horizon are well adapted to degrade easily accessible carbon substrates such as soluble carbohydrates or polysaccharides and, on the contrary, those inhabiting the mineral horizon are better adapted to degrade amino acid derivatives and proteins resulting from the leaching of by-products of organic matter and litter decomposition.

### Carbohydrate-active Enzymes (CAZymes) Screening

**Figure 4:**
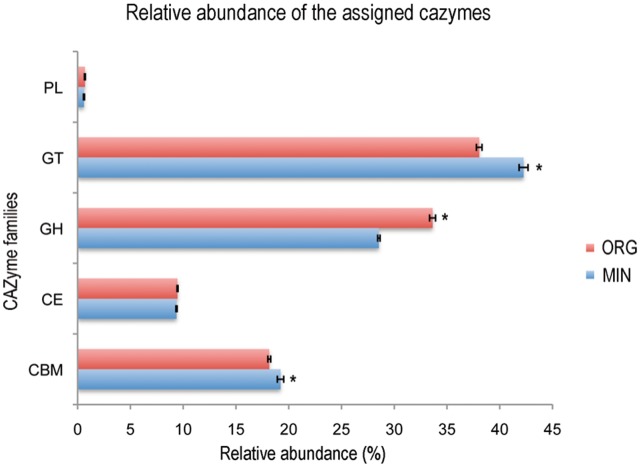
Relative distribution of the sequences encoding putative carbohydrate-active enzymes in the organic and mineral soil horizon metagenomes. A one-factor (habitat) ANOVA was applied on the relative distribution values after an arcsine square root transformation for CAZYME category. Presence of an asterisk indicates that the values are significantly different (p<0.05). PL: polysaccharide lyases, GH: glycoside hydrolase, GT: glycosyl transferase, CE: carbohydrate esterases and CBM: carbohydrate-binding modules.

Due to the importance of organic matter degradation in forest soils and especially the lignocellulose biomass, we focused our analysis on how carbohydrate-active enzymes (CAZymes) coding sequences were distributed in the organic and mineral horizons. Examining the putative CAZyme genes in the pyrosequencing data revealed that significantly more sequences coding for enzymes related to the glycoside hydrolase (GH: glycoside hydrolase; p<0.0001) were detected in the organic horizon (Figure 4). Notably, the mineral horizon was characterised by significantly more enzymes involved in the transfer (GT: glycosyl transferase; p = 0.001) and binding (CBM: carbohydrate-binding modules; p = 0.031) of the glycosides and their derivatives. A total of 260 CAZyme types were identified in the pyrosequencing data, revealing that the 10 most abundant CAZymes represented about 50% of the CAZymes detected (Table S2). A detailed analysis highlighted that 21 GHs, characterised by their ability to hydrolyse substrates such as pectin, cellulose, hemicellulose xylan and glucans, were significantly more abundant in the organic horizon (Table S3). The differences observed in the distribution of the GT and CBM enzymes were mainly due to differences in the enzymes involved in the fixation to cellulose or peptidoglycan or to the synthesis of long-term energy storage compounds such as trehalose or glycogen. Together, these results confirmed the functional sequence analysis performed on MG-RAST and supported the enzymatic and metabolic assays obtained from the soil solutions. These results are consistent with a specialisation of the microbiome inhabiting the organic horizon to the degradation of complex carbohydrates molecules.

### Illumina Contribution to Soil Metagenome Characterisation

The mgDNA samples sequenced by 454 pyrosequencing were also sequenced using the Illumina sequencing. Although the assignment of reads with a size of approximately 75 bp was complex, both the taxonomic and functional analyses were performed using MG-RAST and the SEED database with the default parameters (Table 4). In contrast to the 454 sequences, only 25% of the Illumina sequences had a significant match in the databases, which corresponded to a total of 18.5×10^6^ reads from the Min-S samples and 29.2×10^6^ reads from the Org-S samples. Comparative analysis of the two soil horizons based on the replicates also revealed that 8 and 6 functional categories (Level 1 of MG-RAST) were significantly more abundant in the organic and in the mineral horizon (p<0.005), respectively (Figure 3B). Altogether, these results provided a similar view of the taxonomic and functional distribution of the microbial communities using two different sequencing technologies, demonstrating that this distribution was not linked to the known sequencing bias related to 454 pyrosequencing [59].

**Table 4:** Summary of Illumina data obtained from the soil horizons.

	Mineral horizon			Organic horizon		
	M1	M2	M3	O1	O2	O3
Initial number of reads (75 pb)	28,545,299	33,010,890	31,541,438	35,616,572	33,008,852	32,029,180
Number of reads after cleaning*	23,337,406	26,959,526	25,734,496	29,314,843	27,056,862	26,444,433
Kmer length used for assembly	57	51	51	59	59	59
Number of reads used for assembly	24,245	33,861	22,573	65,718	29,737	43,649
Number of contig	628	945	586	81	516	736
Total assembly length (bp)	144,445	184,219	113,897	28,097	125,141	178,790
Minimum contig length (bp)	113	101	101	117	117	117
Maximum contig length (bp)	1 170	1 204	863	1 968	1 004	1 445
Average contig length (bp)	230	195	194	347	243	243

* Raw sequences were cleaned with fastq_quality_filter program of FASTX-Toolkit package version 0.013 (http: //hannonlab.cshl.edu/fastx_toolkit/index.html).

### Relationship between Metabolic Potentials, Functional and Taxonomic Diversity

Our metagenomic datasets highlight a significant increase of the metabolic diversity (H’_m_; O>M, P = 0.01) and functional diversity (H’_F_; O>M, P = 0.01) in the organic horizon compared to the mineral horizon. Although some differences in the taxa distribution were observed for the bacteria, archaea or eukaryotes, the diversity indices were not significantly different, suggesting that the communities were taxonomically quite similar between the two soil horizons and with a similar diversity, but differentiated at the functional level. Notably, Will *et al*. [9] and Jumpponen *et al*. [47] reported that the diversity indices decreased with depth in a grassland soil, suggesting that this diversity was linked to the microbial biomass, which is known to decrease with soil depth. In our study, quantification of the total bacterial and fungal communities using real time PCR confirmed a decrease of the community densities from the organic to the mineral horizon of the soil, but according to the metagenomic analyses the taxonomic diversity was comparable. Moreover, the differentiation of the horizons according to their H’ indices (H_m_ and H_F_) was associated with a significant correlation between these metabolic and functional diversity indices as confirmed by a linear regression analysis (y = −63.007+22.557x, R^2^ = 0.84 and P = 0.01). Interestingly, a Mantel test performed on the Biolog and metagenomic data also highlighted that a significant correlation existed between these data, suggesting a good correspondence between the metabolic potentials measured on the soil suspensions and the functional metagenomic categories assigned after sequencing (Table 5). The other comparisons presented a good correlation but were not statistically significant (Table 5).

**Table 5:** Pearson correlation (r values), determined via Mantel tests, relating to Euclidean distances dissimilarity matrices generated from scaled biolog, metagenomic functions and taxonomy data.

	Mineral	Organic	Both
Metabolic profile, Functional metagenomic	0.95	0.75	0.703**
Metabolic profile, Phylogenetic metagenomic	0.98	0.62	0.117
Functional metagenomic, Phylogenetic metagenomic	0.99	0.98	0.391

** p-value <0.005.

### Conclusions

Our study presents the first metagenomic characterisation of the soil microbiome using a combination of pyrosequencing and Illumina-based technologies. Analysis of the ca 14 Gbp generated from the forest soil experimental site of Breuil-Chenue confirmed the high complexity of the soil environment and highlighted, at least in the assigned sequences, the prevalence of bacteria in our soil samples. Significant horizon-specific enrichment of some taxa was observed and the communities had significantly different functional abilities. Notably, both the Illumina and 454 sequences gave similar conclusions and highlighted the predominance of Acidobacteria and Alphaproteobacteria in the forest soil. The enrichment of some taxa in the organic or mineral horizon supports the hypothesis of a functional specialization and an important ecological role of these taxa in soil functioning. However, many of the horizon-enriched taxa such as Verrucomicrobia, Bacteriodetes, Chloroflexi or the different phyla of Achaea remain poorly characterized at physiological and functional levels, justifying future research to obtain a comprehensive view of their role. Aside from this relative taxonomic homogeneity, our results suggest that the natural differences existing between the two soil horizons in term of nutrient availability and pH trigger a functional specialisation of the microbial communities. This specialisation was first revealed by the functional assays performed on the soil solutions, was confirmed by the SEED- and CAZymes-based analyses, and showed that the microbial communities inhabiting the organic horizon were significantly enriched for the genes involved in the degradation of soluble carbohydrates and polysaccharides, while those inhabiting the mineral horizon were significantly enriched for the genes involved in the access to carbon derivatives and amino acids. Notably, a Mantel test highlighted a significant correlation between the metabolic potentials (Biolog) and the metagenomic functions only when the organic and mineral horizons were considered together, confirming the quantitative differences existing between the two soil horizon microbiomes. The next step would be to test if a vertical stratification of gene expression exists in the soil using a metatranscriptomic approach. Together, our results demonstrated that the shotgun metagenomic based approach is a valuable tool for assessing microbial functional diversity. Although we are far from obtaining a complete understanding of the soil microbiome, our approach of combining 454 and Illumina data for soil and functional analysis has permitted us to observe the functional specificities of the soil microbial communities inhabiting the organic and mineral horizons. Together, with the recent results of Fierer *et al*. [4], our study illustrates how the natural soil microbial communities are influenced by the soil resource availability, the pH and how they may participate in nutrient cycling.

## Supporting Information

Table S1 Summary of the enzymatic assays performed on soil solutions from the organic and mineral horizons.
(DOC)

Table S2 Average distribution of the CAZYMES detected in the organic and mineral horizons. The table presents the relative distribution (%) of the sequences assigned in CAZYMES categories (GT, CBM, GH, PL and CE).
(XLSX)

Table S3 CAZYME categories presenting significant differences between the two soil horizons. The table presents the relative distribution (%) of the sequences assigned in CAZYMES categories (GT, CBM, GH, PL and CE). The potential function of each CAZYME category is presented.
(XLSX)
