# Supplementary material for: Functional Assays and Metagenomic Analyses Reveals Differences between the Microbial Communities Inhabiting the Soil Horizons of a Norway Spruce Plantation
Source: PLoS One. 2013 Feb 13;8(2):e55929. doi: 10.1371/journal.pone.0055929 (PMC3572175; doi:10.1371/journal.pone.0055929)
Supplement: Table S1 — Summary of the enzymatic assays performed on soil solutions from the organic and mineral horizons. (DOC) [file pone.0055929.s001.doc]

**Table S1:** Summary of the enzymatic assays performed on soil solutions from the organic and mineral horizons.

|  | **Soil horizon** | |  |
| --- | --- | --- | --- |
| **Soil solution** | **Organic (AV)*** | **Mineral (AV)** | statistics |
| ***Ecoplate based tests*** |  |  |  |
| Pyruvic acid methyl ester | 1.02±0.14 | 0.47±0.04 | O>M, *P*=0.009 |
| Tween 40 | 0.90±0.02 | 0.60±0.06 | O>M, *P*=0.004 |
| Tween 80 | 0.76±0.05 | 0.61±0.06 | ns |
| α-cyclodextrin | 0.02±0.004 | 0.01±0.007 | ns |
| glycogen | 0.03±0.004 | 0.02±0.006 | ns |
| D-Cellobiose | 0.28±0.1 | 0.01±0.005 | O>M, *P*=0.045 |
| α-D-Lactose | 0.75±0.4 | 0.002±0.002 | ns |
| B-Methyl-D-glucoside | 1.51±0.32 | 0.27±0.24 | O>M, *P*=0.02 |
| D-Xylose | 0.72±0.23 | 0.01±0.003 | O>M, *P*=0.02 |
| L-Erythritol | 0.18±0.03 | 0.004±0.002 | O>M, *P*=0.0029 |
| D-Mannitol | 1.55±0.09 | 0.92±0.1 | O>M, *P*=0.004 |
| N-Acetyl-D-Glucosamine | 1.68±0.15 | 0.57±0.25 | O>M, *P*=0.01 |
| D-Glucosaminic acid | 1.11±0.17 | 0.16±0.05 | O>M, *P*=0.0019 |
| Glucose-1-phosphate | 0.78±0.28 | 0.12±0.11 | O>M, *P*=0.007 |
| D,L-α-Glycerol phosphate | 0.39±0.05 | 0.12±0.1 | ns |
| D-galactonic acid γ-lactone | 1.00±0.04 | 0.28±0.11 | O>M, *P*=0.0012 |
| D-galacturonic acid | 1.51±0.05 | 0.05±0.04 | O>M, *P*=0.0001 |
| 2-Hydroxy benzoic acid | 0.004±0.003 | 0.0045±0.004 | ns |
| 4-Hydroxy benzoic acid | 0.13±0.03 | 0.03±0.01 | O>M, *P*=0.043 |
| γ-hydroxybutyic acid | 0.16±0.13 | 0.0049±0.003 | ns |
| Itaconic acid | 1.23±0.11 | 0.16±0.05 | O>M, *P*=0.0001 |
| α-Ketobutyric acid | 0.02±0.006 | 0.04±0.01 | ns |
| D-Malic acid | 0.50±0.33 | 0.0006±0.0005 | ns |
| L-arginine | 0.68±0.07 | 0.25±0.06 | O>M, *P*=0.0044 |
| L-asparagine | 1.83±0.11 | 0.85±0.12 | O>M, *P*=0.0014 |
| L-Phenylalanine | 0.09±0.005 | 0.03±0.008 | O>M, *P*=0.001 |
| L-serine | 1.57±0.24 | 0.45±0.06 | O>M, *P*=0.0045 |
| L-threonine | 0.03±0.007 | 0.01±0.003 | O>M, *P*=0.025 |
| Glycyl-L-Glutamic acid | 0.15±0.04 | 0.03±0.02 | O>M, *P*=0.046 |
| Phenylethyl-amine | 0.36±0.17 | 0.01±0.006 | ns |
| Putresceine | 0.46±0.09 | 0.24±0.1 | ns |
| ***Enzymatic tests*** |  |  |  |
| Laccase | 0.80±0.016 | 0.08±0.003 | O>M, *P*=0.0041 |
| Phosphatase | 78210±2214 | 4221±449 | O>M, *P*=0.0004 |
| Glucosidase | 10358±310 | 1225±207 | O>M, *P*=0.0024 |
| Exochitinase | 5820±139 | 512±61 | O>M, *P*=0.0001 |
| Xylosidase | 11181±1070 | 2424±254 | O>M, *P*=0.003 |
| Cellobiohydrolase | 1615±182 | 374±64 | O>M, *P*=0.0038 |

* AV means absorbance values measured for each enzymatic assay at the appropriate OD (see Material and methods section). A one factor (soil horizon) ANOVA was applied on the absorbance values obtained in the assays and results are presented in the column entitled ‘statistics. Ns: non-significant differences. The enzymatic activity for which a significant effect of the soil horizon was observed are presented. O>M means significantly more efficient in the organic (O) horizon than in the mineral (M) horizon.
